# Liver-specific deletion of the *Plpp3* gene alters plasma lipid composition and worsens atherosclerosis in apoE^−/−^ mice

**DOI:** 10.1038/srep44503

**Published:** 2017-03-14

**Authors:** Marco Busnelli, Stefano Manzini, Mika Hilvo, Cinzia Parolini, Giulia S. Ganzetti, Federica Dellera, Kim Ekroos, Minna Jänis, Diana Escalante-Alcalde, Cesare R. Sirtori, Reijo Laaksonen, Giulia Chiesa

**Affiliations:** 1 Department of Pharmacological and Biomolecular Sciences, Università degli Studi di Milano, Milano, Italy; 2 Zora Biosciences Oy, Espoo, Finland; 3 Department of Physiology, Institute of Biomedicine, University of Turku, Turku, Finland; 4 Instituto de Fisiología Celular, División de Neurociencias Universidad Nacional Autónoma de México, Cd. Mx. 04510, México

## Abstract

The *PLPP3* gene encodes for a ubiquitous enzyme that dephosphorylates several lipid substrates. Genome-wide association studies identified *PLPP3* as a gene that plays a role in coronary artery disease susceptibility. The aim of the study was to investigate the effect of *Plpp3* deletion on atherosclerosis development in mice. Because the constitutive deletion of *Plpp3* in mice is lethal, conditional *Plpp3* hepatocyte-specific null mice were generated by crossing floxed *Plpp3* mice with animals expressing Cre recombinase under control of the albumin promoter. The mice were crossed onto the athero-prone apoE^−/−^ background to obtain Plpp3^f/f^apoE^−/−^Alb-Cre^+^ and Plpp3^f/f^apoE^−/−^Alb-Cre^−^ offspring, the latter of which were used as controls. The mice were fed chow or a Western diet for 32 or 12 weeks, respectively. On the Western diet, Alb-Cre^+^ mice developed more atherosclerosis than Alb-Cre^−^ mice, both at the aortic sinus and aorta. Lipidomic analysis showed that hepatic *Plpp3* deletion significantly modified the levels of several plasma lipids involved in atherosclerosis, including lactosylceramides, lysophosphatidic acids, and lysophosphatidylinositols. In conclusion, *Plpp3* ablation in mice worsened atherosclerosis development. Lipidomic analysis suggested that the hepatic *Plpp3* deletion may promote atherosclerosis by increasing plasma levels of several low-abundant pro-atherogenic lipids, thus providing a molecular basis for the observed results.

Lipid phosphate phosphatases (LPPs) are integral membrane proteins with six transmembrane domains, which display broad substrate specificity and catalyse the dephosphorylation of lipid substrates including phosphatidic acid, lysophosphatidic acid, ceramide 1-phosphate, sphingosine 1-phosphate and diacylglycerol pyrophosphate^1^. They belong to a larger phosphatase/phosphotransferase family that includes both membrane and soluble family members^2^.

The LPP family is composed of three enzymes in mammals: LPP1, LPP2 and LPP3, which are encoded by three independent genes named *PLPP1*, *PLPP2* and *PLPP3*, respectively^3^,^4^. The expression of LPP2 mRNA is found mainly in the brain, pancreas and placenta, whereas LPP1 and LPP3 mRNA expression is ubiquitous^5^.

Although the three enzymes demonstrate overlapping catalytic activities and substrate preferences, selective targeted inactivation of the *Plpp* genes in mice indicates that the enzymes have non-redundant functions^6^. In fact, whereas gene inactivation of *Plpp1*/LPP1 or *Plpp2*/LPP2 does not result in any observable phenotype^7^,^8^, *Plpp3*/LPP3 targeted inactivation causes profound developmental defects, thus indicating the essential role of LPP3 in mouse embryonic development^9^.

Interest in *PLPP3*/LPP3 has recently been raised by the results of genome-wide association studies (GWAS)^10^,^11^ that have identified heritable single nucleotide polymorphisms in the *PLPP3* gene and have suggested *PLPP3* as a novel locus associated with coronary artery disease (CAD) susceptibility. Interestingly, the *PLPP3* risk allele independently predicts CAD and lacks associations with traditional risk factors such as hypertension, cholesterol, diabetes mellitus, obesity or smoking.

The generation of a conditional *Plpp3-*null allele^12^ has allowed for the investigation of tissue-specific deletions of the *Plpp3* gene and their effects on vascular health. In previous studies, a lack of *Plpp3* expression in smooth muscle cells has been reported to enhance intimal hyperplasia and vascular inflammation^13^, and more recently, the targeted inactivation of *Plpp3* in endothelial and haematopoietic cells has indicated that LPP3 serves as a negative regulator of endothelial permeability and vascular inflammation^14^.

In the present study, a possible role of *Plpp3* in atherosclerosis development was investigated. The target organ chosen for the selective inactivation of *Plpp3/*LPP3 was the liver, because it is one of the main organs involved in lipid metabolism. Hepatic *Plpp3/*LPP3-deficient mice were then crossed with athero-prone apolipoprotein E knock-out mice apoE^−/−^.

The results indicated that the lack of hepatic *Plpp3* expression increased the levels of several pro-atherogenic plasma lipid species and led to accelerated atherosclerosis progression.

## Results

### Validation of Cre recombinase activity in the liver

**Figure 1:**
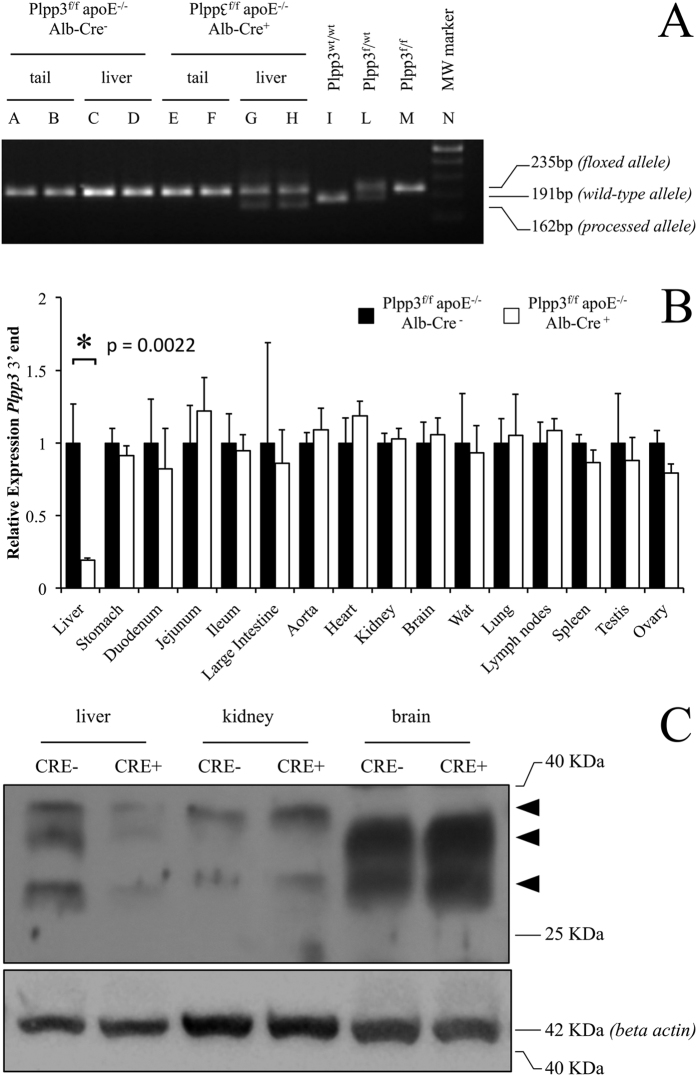
(**A**) Genotyping of the floxed and processed *Plpp3* locus. A representative PCR screening is shown. Tail tips (A-B) and liver tissue (C-D) of Plpp3^f/f^ apoE^−/−^ Alb-Cre^−^ mice show only the 235 bp band corresponding to the floxed, unprocessed *Plpp3* locus. Plpp3^f/f^ apoE^−/−^ Alb-Cre^+^ mice also display the unprocessed *Plpp3* locus band in the tail tips (E-F), but in the liver, both the unprocessed and processed allele (162 bp) bands are present (G-H). Liver PCR amplicons from a wild type Plpp3^wt/wt^ mouse (I), Plpp3^f/wt^ heterozygous mouse (L) and Plpp3^f/f^ parent mouse (M) are shown as controls. Molecular weight marker, 100 bp New England Biolabs (N); (**B**) Quantitative expression of the *Plpp3* mRNA 3′ end. Expression levels of *Plpp3* mRNA are shown, quantified by qPCR analysis detecting a region downstream of the Cre recombinase-mediated floxed exons excision site. The values were normalized to the expression of the transcript in each corresponding tissue of the Plpp3^f/f^ apoE^−/−^ Alb-Cre^−^ mice (n = 6 for liver, n = 3 for all the other tissues; *p = 0.0022 in Plpp3^f/f^ apoE^−/−^ Alb-Cre^+^ vs Plpp3^f/f^ apoE^−/−^ Alb-Cre^−^ mouse liver by Wilcoxon rank-sum test). Wat = white adipose tissue; (**C**) Western blotting analysis of LPP3 expression. A representative image of the immunoblot experiments. Arrowheads indicate the presence of unglycosylated (lower band, 29 KDa) and glycosylated (upper bands, 33–38 KDa) forms of LPP3. The results were confirmed in three biological replicates. Western blotting indicated a substantial decrease in LPP3 in the livers of Plpp3^f/f^ apoE^−/−^ Alb-Cre^+^ mice compared with Plpp3^f/f^ apoE^−/−^ Alb-Cre^−^ mice. In contrast, LPP3 levels remained unchanged in the kidney and brain.

To test the Cre recombinase expression specificity and activity, the *Plpp3* locus was preliminarily examined in the genomic DNA extracted from liver and tail samples of which the latter served as a reference non-target tissue. Genomic DNA extracted from the tails of both Plpp3^f/f^ apoE^−/−^ Alb-Cre^−^ and Plpp3^f/f^ apoE^−/−^ Alb-Cre^+^ animals showed only a PCR band corresponding to the floxed, unprocessed locus (Fig. 1A, lanes A-B, E-F). In contrast, the Alb-Cre transgene was active in the liver of Plpp3^f/f^ apoE^−/−^ Alb-Cre^+^ mice, where the processed allele was detected together with the floxed, unprocessed allele (Fig. 1A, lanes G-H). As expected, the *Plpp3* locus was unprocessed in the liver of Plpp3^f/f^ apoE^−/−^ Alb-Cre^−^ animals (Fig. 1A, lanes C-D).

### Plpp3^f/f^ apoE^−/−^ Alb-Cre^+^ mice exhibit markedly reduced hepatic LPP3 expression, as detected via qPCR and Western blotting

To investigate the effect of Cre^−^mediated *Plpp3* locus recombination on *Plpp3* transcription, two quantitative PCR primer sets were designed, targeting the 5′ (upstream of the Cre recombinase mediated excision of crucial *Plpp3* exons) and 3′ end (located within the DNA sequences excised by the Cre recombinase) of *Plpp3* mRNA. This approach was able to evaluate *Plpp3* promoter activity, by probing *Plpp3* mRNA expression upstream of the site of action of Cre recombinase in the genome (5′ end primer pair) and the functional effect of Alb-Cre^−^mediated recombination, which results in truncated and non-functional *Plpp3* mRNA (3′ end primer pair).

The Cre recombinase had no effect on the 5′ end of *Plpp3* mRNA in both mouse lines; both Plpp3^f/f^ apoE^−/−^ Alb-Cre^−^ and Plpp3^f/f^ apoE^−/−^ Alb-Cre^+^ tissues showed comparable expression levels (see Supplementary Figure S1). In contrast, quantitative PCR using the 3′ end primer pair showed a marked decrease of *Plpp3* mRNA expression in the livers of Plpp3^f/f^ apoE^−/−^ Alb-Cre^+^ mice compared to the livers of Plpp3^f/f^ apoE^−/−^ Alb-Cre^−^ mice (Fig. 1B). No relevant differences were observed between the two genotypes in all the other tissues analysed (Fig. 1B). A major liver-specific decrease of LPP3 protein expression in Plpp3^f/f^ apoE^−/−^ Alb-Cre^+^ mice was also confirmed by Western blotting analysis (Fig. 1C).

### Hepatic *Plpp3* deletion does not affect the hepatic parenchyma and lipid deposition

The hepatic parenchyma was unaffected by liver-specific *Plpp3* deletion; haematoxylin and eosin (H&E) staining showed no differences in the livers from Plpp3^f/f^ apoE^−/−^ Alb-Cre^−^ and Plpp3^f/f^ apoE^−/−^ Alb-Cre^+^ mice fed chow diet for 32 weeks starting after weaning (8 weeks of age) (Supplementary Figure S2). The Western diet, which was administered for 12 weeks starting after weaning, increased lipid accumulation in both genotypes but resulted in no significant differences between the two mouse lines. Specifically, the percentage of Oil Red O positive area over the total area was 77.4 ± 2.1% in Plpp3^f/f^ apoE^−/−^ Alb-Cre^−^ and 80.3 ± 5.2% in Plpp3^f/f^ apoE^−/−^ Alb-Cre^+^ mice, n = 10, p > 0.05 (Supplementary Figure S2). In addition, van Gieson and Periodic acid-Schiff (PAS) staining did not reveal any differences in collagen and glycogen deposition between Plpp3^f/f^ apoE^−/−^ Alb-Cre^+^ and Plpp3^f/f^ apoE^−/−^ Alb-Cre^−^ mice (Supplementary Figure S2).

### Plpp3^f/f^ apoE^−/−^ Alb-Cre^+^ mice on a Western diet display an enlargement of atherosclerotic plaques driven by an enlarged necrotic core

To investigate the role played by hepatic LPP3 during atherosclerosis development, Plpp3^f/f^ apoE^−/−^ Alb-Cre^−^ and Plpp3^f/f^ apoE^−/−^ Alb-Cre^+^ mice were fed, after weaning, a chow diet for 32 weeks or a Western diet for 12 weeks.

**Figure 2:**
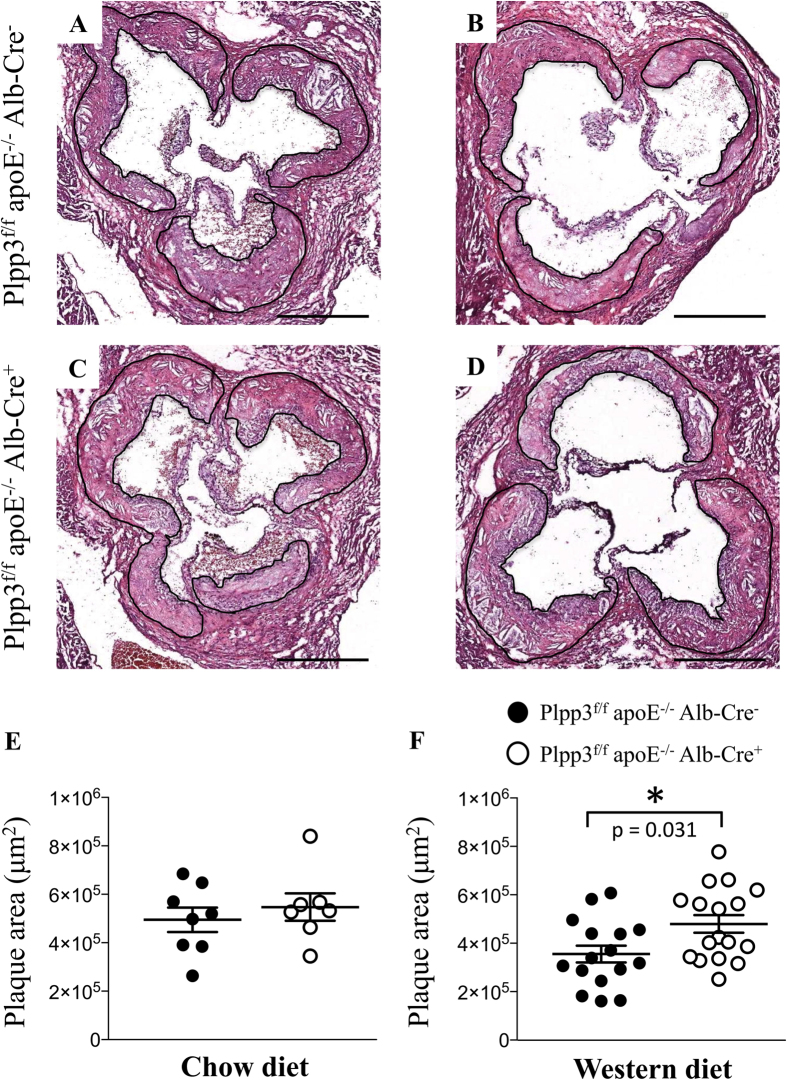
Representative H&E photomicrographs and quantification of the maximum plaque area at the aortic sinuses in Plpp3^f/f^ apoE^−/−^ Alb-Cre^+^ and Plpp3^f/f^ apoE^−/−^ Alb-Cre^−^ mice. The atherosclerosis development in the chow fed mice (n = 7–8) was comparable between the two genotypes (**A**,**C**,**E**). Western diet administration significantly worsened atherosclerosis development in Plpp3^f/f^ apoE^−/−^ Alb-Cre^+^ compared with Plpp3^f/f^ apoE^−/−^ Alb-Cre^−^ mice (n = 16–17) (**B**,**D**,**F**). The data are shown as the mean ± SEM; *p = 0.031 vs Plpp3^f/f^ apoE^−/−^ Alb-Cre^−^ by Wilcoxon rank-sum test. Bar length = 500 μm.

**Figure 3:**
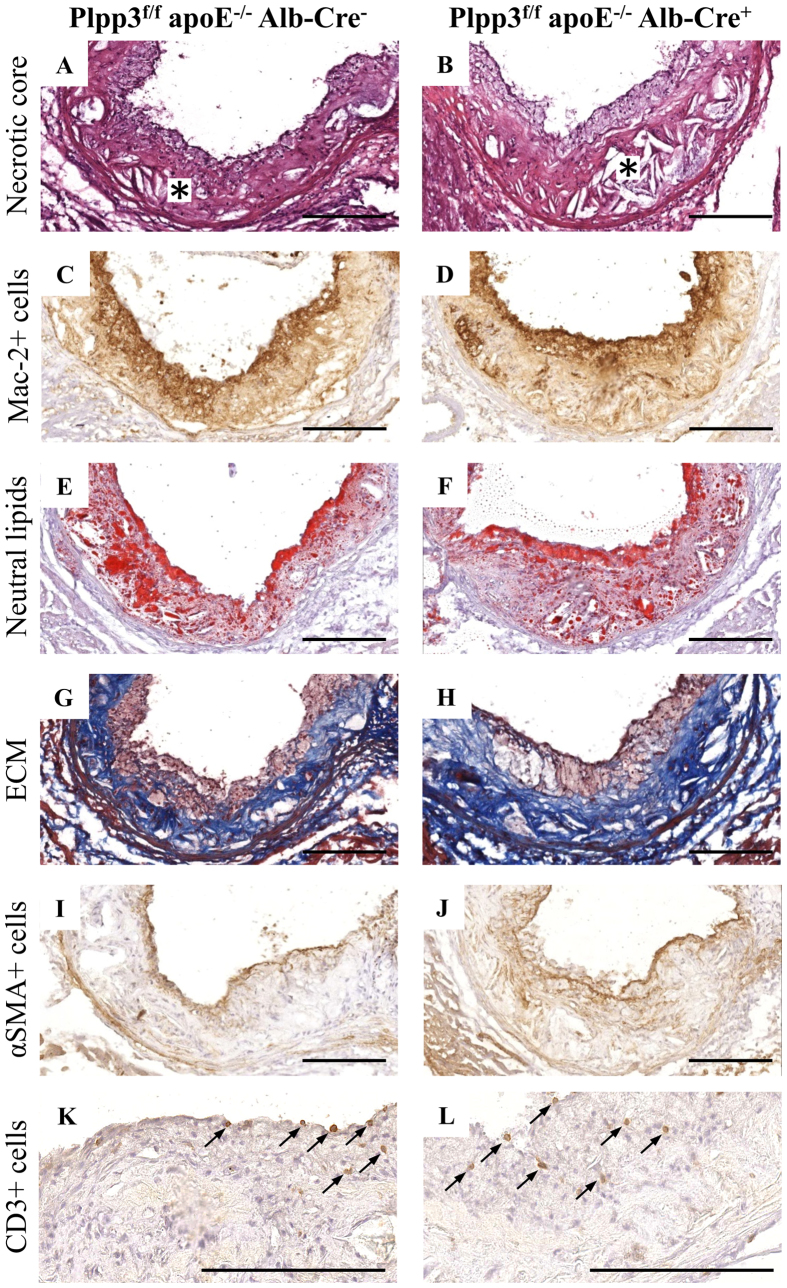
Histological and immunohistochemical characterization of plaques at the aortic sinuses in Plpp3^f/f^ apoE^−/−^ Alb-Cre^−^ and Plpp3^f/f^ apoE^−/−^ Alb-Cre^+^ mice fed a Western diet. Representative photomicrographs of the necrotic core (**A**,**B**), macrophages (Mac-2 + cells; **C**,**D**), neutral lipids (Oil Red O; **E**,**F**), extracellular matrix (ECM; **G**,**H**), smooth muscle cells (αSMA + celIs; **I**,**J**) and CD3 + cells (**K**,**L**). Bar length = 200 μm. Asterisks mark the localization of the necrotic core (**A**,**B**).

No differences in both plaque size and composition were observed at the aortic sinuses in the two mouse lines fed the chow diet (Fig. 2A,C,E and Table 1). In contrast, when challenged with a Western diet, Plpp3^f/f^ apoE^−/−^ Alb-Cre^+^ mice showed a 35% increase in atherosclerosis development, as compared with Plpp3^f/f^ apoE^−/−^ Alb-Cre^-^ mice (p = 0.031) (Fig. 2B,D,F and Table 1). This increase appeared to be primarily attributable to a larger necrotic core area (Table 1 and Fig. 3). A trend towards increased accumulation of extracellular matrix (ECM) was observed in Plpp3^f/f^ apoE^−/−^ Alb-Cre^+^ mice (p = 0.063). No significant variations were found in neutral lipid deposition, macrophages and smooth muscle cell accumulation (detected with Oil Red O, Mac-2 and αSMA staining, respectively). These variations in the plaque components resulted in a higher percentage of the necrotic core and a concomitantly decreased percentage of neutral lipids and macrophages (Table 1 and Fig. 3).

**Figure 4:**
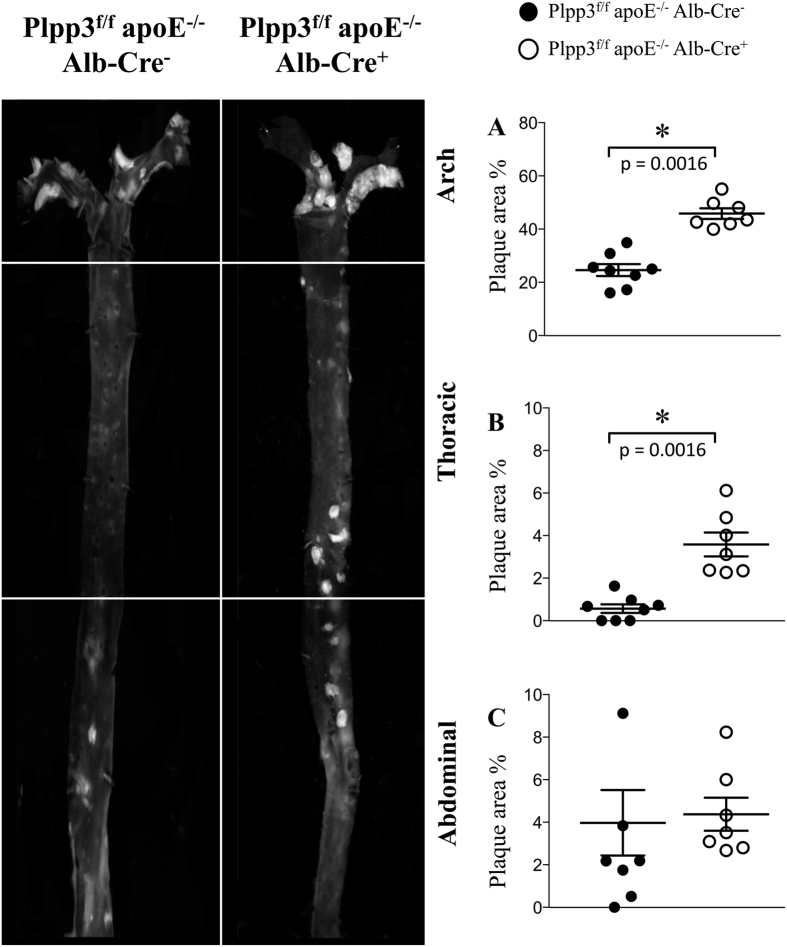
Representative photomicrographs and quantification of plaque area detected by en-face analysis of the aortas of mice fed a Western diet. After 12 weeks of dietary treatment, whole aortas were collected and en-face analysis was performed to quantify the percentage of aortic surface covered by atherosclerotic plaques. Atherosclerotic plaque development in the arches (**A**) and in the thoracic segments (**B**) was significantly increased in the aortas from Plpp3^f/f^ apoE^−/−^ Alb-Cre^+^ mice (white dots) compared with Plpp3^f/f^ apoE^−/−^ Alb-Cre^−^ mice (black dots). Atherosclerosis development at the abdominal segment (**C**) was not different between the two genotypes. Data are shown as the mean ± SEM; n = 7–8 mice per group, p = 0.0016 in the arch and p = 0.0016 in the thoracic segment vs Plpp3^f/f^ apoE^−/−^ Alb-Cre^−^ mice by Wilcoxon rank-sum test.

En-face analysis of the entire aorta of the Western diet-fed mice showed a dramatic increase in aortic atherosclerosis in Plpp3^f/f^ apoE^−/−^ Alb-Cre^+^ mice, specifically in the aortic arch and the thoracic segment, as compared with Plpp3^f/f^ apoE^−/−^ Alb-Cre^−^ mice (Fig. 4A,B). No differences in atherosclerosis burden were observed in the abdominal aortas of either genotype (Fig. 4C).

### Plpp3^f/f^ apoE^−/−^ Alb-Cre^+^ mice on a Western diet exhibit increased levels of plasma triglycerides

On a chow diet, Plpp3^f/f^ apoE^−/−^ Alb-Cre^+^ and Plpp3^f/f^ apoE^−/−^ Alb-Cre^−^ mice did not show significant differences in circulating total-cholesterol (TC) and HDL-cholesterol (HDL-C) levels, as well as in plasma triglycerides (TAG) and phospholipids (PL) (TC: 296.4 ± 47.3 vs 271.5 ± 53.1 mg/dl, respectively; HDL-C: 14.3 ± 2.7 vs 15.0 ± 4.8 mg/dl, respectively; TAG: 47.7 ± 9.6 vs 53.0 ± 13.8 mg/dl, respectively; PL: 231.1 ± 34.3 vs 228.2 ± 46.1 mg/dl, respectively; n = 10, p > 0.05). In contrast, when the mice were administered a Western diet, a significant increase of TAG was observed in Plpp3^f/f^ apoE^−/−^ Alb-Cre^+^ vs Plpp3^f/f^ apoE^−/−^ Alb-Cre^−^ mice (TAG: 301.1 ± 103.0 vs 162.4 ± 48.3 mg/dl, respectively, p = 0.0012). A trend towards an increase in plasma PL levels was also observed in Plpp3^f/f^ apoE^−/−^ Alb-Cre^+^ (703.9 ± 106.6 vs 588.6 ± 103.7 mg/dl, respectively, n = 10, p = 0.054). No significant differences were observed for TC levels (939.2 ± 190.5 vs 899.5 ± 218.8 mg/dl, respectively, p > 0.05).

### Hepatic Plpp3 deletion results in an increased concentration of lysophosphatidic acids, lactosylceramides and several lysophospholipids in plasma

To gain insights into changes caused by *Plpp3* deletion in plasma lipid levels, lipidomic analysis was performed using global lipidomics methods as well as targeted ceramide and lysophospholipid platforms. The statistical results for all lipids and comparisons together with q-values controlling the false discovery rate are presented in Supplementary Data S1. The Western diet, compared with chow diet, increased the levels of most lipid classes in both mouse lines (see Supplementary Table S1). The effects of hepatic *Plpp3* deficiency on the plasma lipidome in mice fed chow or Western diets is shown in Tables 2 and 3 as well as Supplementary Figure S3. The hepatic *Plpp3* deletion increased the levels of lactosylceramides (LacCer) in mice on both the chow and Western diets. In addition, on Western diet, the levels of lysophosphatidic acids (LPA) and lysophosphatidylinositols (LPI) were significantly increased in Plpp3^f/f^ apoE^−/−^ Alb-Cre^+^ vs Plpp3^f/f^ apoE^−/−^ Alb-Cre^−^ mice (Table 2). Globotriaosylceramides (Gb3) were also increased, although this variation did not reach statistical significance (p = 0.055).

Table 3 and Supplementary Figure S3 show the molecular lipids that were significantly altered by hepatic *Plpp3* deletion in mice fed chow or a Western diet. Several LacCer lipids were significantly increased in Plpp3^f/f^ apoE^−/−^ Alb-Cre^+^ mice on both diets. LPAs, lysophosphatidylethanolamines (LPEs) and LPIs were also increased with both dietary treatments, but reached statistical significance only with the Western diet. In these lipid classes, the fatty acids varied from short-saturated to long-polyunsaturated fatty acids, thus indicating that the alterations were due to broad changes of these lipid classes rather than to alterations in individual fatty acids.

## Discussion

On the basis of the results of GWAS that associated the *PLPP3* gene polymorphisms with CAD susceptibility^10^,^11^, a possible role of *Plpp3*/LPP3 in atherosclerosis development was investigated in a genetically modified mouse model. The function of LPP3, the enzyme encoded by *Plpp3*, is that of dephosphorylating lipid substrates, and therefore, the liver was chosen as the target organ for *Plpp3* deletion, because the liver is among the main sources of circulating plasma lipids and lipoproteins, which significantly contribute to the atherosclerosis process. It is well known that, during atherogenesis, LDLs enter the arterial wall, either oxidized or enzymatically degraded, and follow the atherogenic pathway that, through the involvement of macrophages and smooth muscle cells, leads to cholesterol deposition and atherosclerotic plaque formation^15^. Importantly, the lipoproteins responsible for cholesterol transport, mainly LDLs and HDLs, also contain hundreds of other associated lipid species that may exhibit various bioactive properties that affect the course of the disease^16^. Among those, LPP3 lipid substrates or their precursors have been identified as components of the circulating lipoproteins^17^.

The liver-specific deletion of *Plpp3* was achieved by crossing *Plpp3*-floxed mice with animals expressing Cre recombinase under the control of the hepatocyte-specific albumin promoter. In the livers of Plpp3^f/f^ apoE^−/−^ Alb-Cre^+^ mice, compared with Cre recombinase negative mice, the *Plpp3* expression levels decreased over five-fold. The presence of a residual *Plpp3* functional gene in liver, also demonstrated via PCR analysis of liver genomic DNA, may be explained by the presence of non-parenchymal, i.e. non-hepatocyte, cell types, such as sinusoidal endothelial cells, Kupffer cells, hepatic stellate cells and, often, intrahepatic lymphocytes^18^,^19^. It should be noted that these cells, which do not express albumin^20^, contribute to only 6.5% of the liver volume, but account for approximately 40% of the total number of liver cells^19^. Comparable expression levels were observed in all the other organs/tissues assayed in the Plpp3^f/f^ apoE^−/−^ Alb-Cre^+^ and Plpp3^f/f^ apoE^−/−^ Alb-Cre^−^ animals, thus demonstrating the high specificity of the albumin promoter expression and Cre recombinase activity.

The main result of the present manuscript is the demonstration that hepatocyte-specific *Plpp3* deletion is associated with increased atherosclerosis progression. This result was observed when mice were fed Western diet (+35% plaque area at the aortic sinus and +86% plaque extent at the aortic arch of Alb-Cre^+^ vs Alb-Cre^−^ mice), whereas no differences were found in atherosclerosis development between the two genotypes, when mice were fed a chow diet. The plaque composition was not different between the two mouse lines on a chow diet; however, under a Western diet, a larger necrotic core was observed in Plpp3^f/f^ apoE^−/−^ Alb-Cre^+^ mice. Interestingly, no differences in the main circulating plasma lipid levels were found in chow fed animals, whereas Western diet-fed Plpp3^f/f^ apoE^−/−^ Alb-Cre^+^ mice displayed increased TAG plasma levels vs Plpp3^f/f^ apoE^−/−^ Alb-Cre^−^ mice.

Because Lpp3 is a lipid phosphatase with a large number of substrates, specific investigations into all the individual Lpp3 targets would not be possible. Thus, with the aim of elucidating the effects of hepatic *Plpp3* deletion on circulating lipid levels, which might affect atherosclerosis development, a lipidomic analysis was conducted on Plpp3^f/f^ apoE^−/−^ Alb-Cre^−^ and Plpp3^f/f^ apoE^−/−^ Alb-Cre^+^ mouse plasma. Statistical analyses of the lipidomics data revealed that none of the lipids were significantly different after multiple hypothesis correction. However, significant non-adjusted p-values for several lipids in the same or related lipid classes were observed, particularly in mice on the Western diet. Therefore, we consider these results in mice on the Western diet to be biologically relevant.

Comparison of Plpp3^f/f^ apoE^−/−^ Alb-Cre^−^ and Plpp3^f/f^ apoE^−/−^ Alb-Cre^+^ mice fed either a chow diet or a Western diet indicated a significant increase in LacCer in Plpp3^f/f^ apoE^−/−^ Alb-Cre^+^ mice. A direct link between LacCer accumulation and *Plpp3* deletion is difficult to establish, given the complexity of the biosynthetic routes that convert ceramide to other bioactive sphingolipids in mammalian cells^21^. However, a recent study with apoE^−/−^ mice, although in a different experimental setting, has reported an increase in the neutral glycosphingolipid LacCer when *Plpp3* expression is decreased^22^. In animal models, increased levels of LacCer and other sphingolipids have been associated with the development of atherosclerosis^23^,^24^, and several enzymes in the glycosphingolipid synthesis pathway have been tested as potential anti-atherosclerotic drug targets^25^,^26^. Elevated sphingolipid levels in human plasma have also been indicated as a risk factor for atherosclerosis development^27^. Interestingly, in a recent clinical study, different sphingolipids and glycosphingolipids, especially LacCer, have been associated with CAD outcome and plaque vulnerability, which was identified on the basis of an enlarged necrotic core^28^. This observation supports our results, because in Plpp3^f/f^ apoE^−/−^ Alb-Cre^+^ mice fed a Western diet, the worsening of atherosclerosis was associated with a larger necrotic core. In Plpp3^f/f^ apoE^−/−^ Alb-Cre^+^ mice fed a chow diet, however, the increase in LacCer levels was not associated with increased atherosclerosis development. Together, these observations do not indicate a straightforward link between higher plasma LacCer concentrations and atherosclerosis. However, it cannot be excluded that this plasma lipid perturbation nonetheless contributed to the accelerated atherosclerotic process observed in mice fed the Western diet.

The Western diet, compared with the chow diet, in both mouse lines, led to higher plasma concentrations of cholesteryl esters, sphingolipids and most glycerophospholipids. Interestingly, Plpp3^f/f^ apoE^−/−^ Alb-Cre^+^ mice showed a statistically significant increase in TAG and LPA levels vs Plpp3^f/f^ apoE^−/−^ Alb-Cre^−^ mice only when fed the Western diet.

LPA is a well-known substrate of LPP3, which dephosphorylates and thus terminates LPA’s receptor-mediated signalling actions^29^. Previous studies have demonstrated that the liver plays a major role in LPA catabolism^30^. The increase in LPA observed in Plpp3^f/f^ apoE^−/−^ Alb-Cre^+^ mice fed a Western diet supports these observations and indicates the involvement of hepatic LPP3 in LPA degradation. Moreover, because LPA is an obligate intermediate in TAG synthesis^31^, the increased LPA levels in Plpp3^f/f^ apoE^−/−^ Alb-Cre^+^ mice are consistent with the elevated plasma TAG levels observed. In humans, it has been observed that hyperlipidemia predisposes to the generation of LPA in the circulation^32^, and higher concentrations of serum LPA have been associated with the occurrence of acute coronary syndromes^33^. Moreover, in apoE^−/−^ mice, systemic treatment with unsaturated LPA has been shown to have pro-inflammatory and pro-atherosclerotic effects^34^. In our study, we observed increased circulating amounts of LPA 18:1 in the sn-2 position and 20:4 in the sn-1 position. These LPA molecular species have previously been recognized to have high atherogenic and thrombogenic potency in the lipid-rich cores of human atherosclerotic plaques^35^ as well as to promote dyslipidemia^36^. Interestingly, an association between necrotic core formation and LPA accumulation has been highlighted in previous studies^35^. These findings support a link between the elevated plasma LPA concentration and the increased necrotic core areas observed in the aortic sinus of Plpp3^f/f^ apoE^−/−^ Alb-Cre^+^ mice fed a Western diet.

The Plpp3^f/f^ apoE^−/−^ Alb-Cre^+^ mice fed a Western diet also displayed a significant increase of LPI plasma concentration compared with that in Western-fed Plpp3^f/f^ apoE^−/−^ Alb-Cre^−^ mice. LPI is synthesized by several cell types^37^,^38^,^39^ and is generated by phospholipase A2, which catalyses the hydrolysis of phosphatidylinositol and generates LPI and free arachidonic acid^40^. Most of the biological effects of LPI are mediated by the receptor GPR55^41^. LPI may play a pro-atherogenic role by promoting endothelial dysfunction. Indeed, several studies have indicated that LPI negatively regulates endothelial cells migration and induces VCAM-1 and ICAM-1 expression^42^,^43^.

Finally, Gb3 showed a trend towards increased levels in Plpp3^f/f^ apoE^−/−^ Alb-Cre^+^ mice fed a Western diet. This increase is consistent with the observed elevation of LacCer, because Gb3 is synthesized from LacCer by α1,4-galactosyltransferase^44^. Circulating Gb3 is transported in LDL and HDL particles^45^,^46^. In the arterial wall, Gb3 accumulation leads to extracellular matrix deposition and calcification in the media^47^, whereas, within the endothelium, it promotes the production of reactive oxygen species, upregulates the expression of adhesion molecules^48^, and causes endothelial dysfunction^49^.

In conclusion, the present work provides the first demonstration of the role of *Plpp3*/LPP3 in atherosclerosis development in an animal model and further provides experimental evidence supporting clinical observations relating *PLPP3* polymorphisms to CAD susceptibility. Additionally, plasma lipidomic analysis suggested a molecular basis for the observed results, indicating low-abundant lipid species as potential players in the development of atherosclerosis associated with *Plpp3* hepatic deficiency.

## Methods

Procedures involving animals and their care were conducted in accordance with institutional guidelines that are in compliance with national (D.L. No. 26, March 4, 2014, G.U. No. 61 March 14, 2014) and international laws and policies (EEC Council Directive 2010/63, September 22, 2010: Guide for the Care and Use of Laboratory Animals, United States National Research Council, 2011). The experimental protocol was approved by the Italian Ministry of Health (Protocollo 2012/4 and 434/2016-PR).

### Mouse models

Conditional *Plpp3*/LPP3 liver-specific null mice were generated by crossing mice with the *Plpp3*/LPP3 gene flanked by loxP sites (Plpp3^f/f^), which were kindly provided by Dr. Susan R. Schwab^12^, with mice expressing the Cre recombinase under control of a hepatocyte-specific albumin promoter (Alb-Cre^+^, The Jackson Laboratory, USA). Two oligonucleotides (Cre_a: 5′-AGGTGTAGAGAAGGCACTCAGC-3′, Cre_b: 5′- CTAATCGCCATCTTCCAGCAGG-3′) were used to screen wild-type (no PCR amplicon) and Cre recombinase positive mice (412 bp). The PCR cycling conditions were 95 °C for 4 min, followed by 40 cycles of 30 s at 95 °C, 30 s at 60 °C and 30 s at 72 °C. Three oligonucleotides (fwd_a: 5′-CTACAGATGTCAGTCAGTGTG-3′, fwd_c: 5′-GAAGTGCCATTACTCTCTCAGC-3′ and rev_d: 5′-CCAGGGTGCTATCTATCTGTAAC-3′) were used to screen for wild-type, floxed and recombined *Plpp3* alleles in the genomic DNA (191 bp, 235 bp and 162 bp, respectively), as described elsewhere^12^.

Plpp3^f/f^ mice, hemizygous for Cre recombinase, were crossed onto an athero-prone genetic background through multiple crosses with homozygous apolipoprotein E knock-out (apoE^−/−^) mice (Charles River Laboratories, Italy) to obtain Plpp3^f/f^ apoE^−/−^ Alb-Cre^+^ (lacking Plpp3 hepatic expression) and Plpp3^f/f^ apoE^−/−^ Alb-Cre^−^ (as control) mice. A set of three primers (eko_a: 5′-GCCTAGCCGAGGGAGAGCCG-3′, eko_b: 5′-TGTGACTTGGGAGCTCTGCAGC-3′ and eko_c: 5′-GCCGCCCCGACTGCATCT-3′) was used to screen wild type, apoE^−/−^ and apoE^+/−^ mice, as described elsewhere (http://jaxmice.jax.org/strain/002052.html).

All mice were maintained on a C57Bl/6 background. Plpp3^f/f^ apoE^−/−^ Alb-Cre^−^ and Plpp3^f/f^ apoE^−/−^ Alb-Cre^+^ mice, at 8 weeks of age, were divided into two groups and fed a Western diet (TD.88137, Harlan Laboratories, Italy) for 12 weeks or a regular chow diet (4RF21, Mucedola, Italy) for 32 weeks.

### Plasma and tissue harvesting

At the end of the experimental period, after an overnight fast, blood was collected from the retro-orbital plexus, under 2% isoflurane anaesthesia (Forane, Abbot Laboratories Ltd, Illinois, USA), into tubes containing 0.1% (w/v) EDTA and plasma was separated after centrifugation for 10 min at 5900× g at 4 °C. The mice were then sacrificed under general anaesthesia with 2% isoflurane and the blood was removed by perfusion with phosphate-buffered saline (PBS). The aorta was rapidly dissected from the aortic root to the iliac bifurcation, and as much periadventitial fat and connective tissue were removed as much as possible. The aorta was then longitudinally opened, pinned flat on a black wax surface in ice-cold PBS and photographed unstained^50^,^51^ for plaque quantification (see En face analysis). For histological/immunohistochemical analysis, the hearts were removed, fixed in 10% formalin for 30 min and transferred into PBS containing 20% sucrose (w/v) overnight at 4 °C before being embedded in OCT compound (Sakura Finetek, The Netherlands) and stored at −80 °C.

The apex of the heart, liver, stomach, duodenum, jejunum, ileum, large intestine, kidney, brain, abdominal white adipose tissue, lung, inguinal lymph node, spleen, testis and ovary were immediately snap-frozen in liquid nitrogen for subsequent analyses.

### Quantitative PCR

Total RNA was isolated using a NucleoSpin RNA extraction kit (Macherey–Nagel, Germany) and total RNA (1 μg) was reverse transcribed with random hexamers and MultiScribe reverse transcriptase (Applied Biosystems, California, USA) by following the manufacturer’s instructions.

The expression level of the 5′ end (5p_fw: 5′-ACCGTCGAGGGTTTTACTGC-3′; 5p_rev: 5′-GAGCGGGACTTCTCCTTGAG-3′) and 3′ end (3p_fw: 5′-CACGGGATTGTCACGGGTAT-3′; 5p_rev: 5′-AGGTCGGACACGAAGAACAC-3′) of the murine *Plpp3* gene was quantified by qPCR on a Biorad CFX Connect thermal cycler with iQ SYBR Green Supermix (Biorad, California, USA) in 15 μl reactions, with 300 μM of each primer. The cycling conditions were 95 °C for 4 min, followed by 40 cycles of 30 s at 95 °C, 30 s at 55 °C and 30 s at 72 °C. A final melting curve analysis assured the authenticity of the target product. The housekeeping gene cyclophilin A (*Ppia*) (ppia_fw: 5′-AGCACTGGGGAGAAAGGATT-3′; ppia_rev: 5′-AGCCACTCAGTCTTGGCAGT-3′) was used for normalization.

### Western blotting analysis

Liver, kidney and brain tissue samples were lysed in ice-cold RIPA buffer (50 mM Tris–Cl pH 8.0, 150 mM NaCl, 1% NP-40, 0.5% sodium deoxycholate, 0.1% sodium dodecyl sulphate) supplemented with a 2× Protease Inhibitor Cocktail (Sigma-Aldrich). The lysates were then sonicated and the protein concentration was determined using the BCA protein assay method. A total of 60 μg of proteins was separated by SDS–PAGE in 10% acrylamide gels and transferred to a membrane. The following antibodies were used for Western blotting analysis: a previously validated purified rabbit anti-LPP3 (1:2000, Sigma, custom-made^52^,^53^) generated against a peptide antigen spanning the residues 2–7 of human LPP^1^ and a beta actin monoclonal mouse antibody (clone AC-15, Sigma-Aldrich). The membranes were then washed and further incubated with horseradish peroxidase-conjugated anti-rabbit or anti-mouse IgG (#7074 and #7076 respectively, Cell Signaling, Maryland, USA). The signal was detected with the Pierce ECL Western Blotting Substrate (Thermo Scientific, Massachusetts, USA) and quantified with Image Studio 5.2.5 (Li-Cor, Nebraska, USA).

### Histology

#### Aortic sinus

Serial cryosections (7 micron thick) of the aortic sinus were cut. Approximately 25 slides with 3 cryosections/slide were obtained, spanning the three cusps of the aortic valves. Every sixth slide was stained with haematoxylin and eosin (H&E, Bio-Optica, Italy) to determine the plaque area, which was calculated as the mean area of those sections showing the three cusps of the aortic valves and the necrotic core area, which was defined as the acellular area that was not stained by H&E^54^. The adjacent slides were stained with Oil Red O (Sigma-Aldrich, Missouri, USA) to detect intraplaque neutral lipids and with Masson’s trichrome (Bio-Optica, Milano, Italy) to determine extracellular matrix deposition. Macrophages, smooth muscle cells (SMCs) and T lymphocytes were detected using an anti-Mac2 antibody (CL8942, Cedarlane, Ontario, Canada), an anti-α-smooth muscle actin antibody (Ab5694, Abcam, Cambridge, UK) and an anti-CD3 antibody (MAB4841, R&D Systems, Minnesota, USA), respectively. Detection was performed using an ImmPRESS reagent kit (Vector Laboratories, Peterborough, UK). 3,3′-Diaminobenzidine was used as the chromogen (Sigma-Aldrich), and the sections were counterstained with Gill’s haematoxylin (Bio-Optica). The Aperio ScanScope GL Slide Scanner (Aperio Technologies, California, USA) was used to acquire digital images that were subsequently processed with the ImageScope software. An operator blinded to the dietary treatment and genotype quantified plaque size and composition.

#### Liver

The left lobes were isolated, immersion-fixed in 10% formalin for 24 hours, dehydrated in a graded scale of ethanol, and paraffin embedded. Serial sections (4 micron thick) were cut and stained with H&E. In addition, Periodic acid-Schiff (PAS) staining was performed to evaluate glycogenosis and van Gieson staining was performed to evaluate collagen deposition. Another lobe of the liver was also embedded in OCT and cryosections (7 micron thick) were stained with Oil Red O to detect neutral lipid accumulation. Hepatic lipid deposition was measured as the percentage of Oil Red O positive area over the total area measured by the ImageScope software.

### En Face Analysis

The images of the aorta were recorded with a stereomicroscope-dedicated camera (IC80 HD, MZ6 microscope, Leica Microsystems, Germany) and analysed using the ImageJ image processing program (http://rsb.info.nih.gov/ij/). An operator blinded to the dietary treatment quantified the atherosclerotic plaques.

### Lipid analysis

Plasma TC, TAG and PL were measured with enzymatic methods (ABX Diagnostics, France and B.L. Chimica, Italy). HDL-C levels were measured after the precipitation of apoB-containing lipoproteins with PEG (20% w/v) in 0.2 mol/L glycine (pH 10)^55^.

### Lipidomic analyses

Ceramides and glycerophospholipids were extracted using a modified Folch lipid extraction method^56^, and lysophospholipids were extracted with the butanol extraction method^57^, using a Hamilton Microlab Star robot (Hamilton Robotics, Switzerland). The samples were spiked with known amounts of non-endogenous synthetic internal standards. After lipid extraction, the samples were reconstituted in chloroform:methanol (1:2, v/v) and synthetic external standards were spiked into the extracts post-extraction. The extracts were stored at −20 °C before MS analysis.

In Shotgun Lipidomics, the lipid extracts were analysed using a hybrid triple quadrupole/linear ion trap mass spectrometer (QTRAP 5500, SCIEX, Canada) equipped with a robotic nanoflow ion source (TriVersa NanoMate, NY, USA) according to the methods described by Ståhlman and colleagues^58^. The molecular lipids were analysed in both positive and negative modes by using Multiple Precursor Ion Scanning (MPIS) based methods^59^,^60^. The molecular lipid species were identified and quantified in semi-absolute or absolute amounts^58^. Targeted molecular lipids were analysed on a hybrid triple quadrupole/linear ion trap mass spectrometer (QTRAP 5500) equipped with an ultra-high pressure liquid chromatography (UHPLC) system (Eksigent ekspert 100-XL, SCIEX, Canada) using multiple reaction monitoring (MRM)–based methods in positive and negative ion modes. Regioisomers 1-acyl-2-LPL (sn1) and 2-acyl-1-LPL (sn2) were detected on the basis of their different elution times. The lipids were normalized to their respective internal standard and to the sample amounts. All the lipidomics data are provided as μmol/L in Supplementary Data S2. The raw mass spectrometric data files are available upon request for non-commercial scientific purposes.

### Statistical analyses

Statistical analyses for the lipidomics data were performed using R (Version x64 3.3.2). Wilcoxon rank-sum tests were performed to compare the differences between the study groups. In comparisons, the mean relative differences are presented as relative percentage differences in the mean concentrations of the groups. The results of all statistical analyses for the lipids are presented in Supplementary Data S1, which also shows Q-values which indicate false discovery rate.

Statistical analyses for all other comparisons were performed using the GraphPad Prism version 7.0b for Mac, GraphPad Software, La Jolla California USA, www.graphpad.com and the group differences were tested for statistical significance with Wilcoxon rank-sum test. In the statistical analyses, p < 0.05 was considered significantly different.

## Additional Information

**How to cite this article**: Busnelli, M. *et al*. Liver-specific deletion of the *Plpp3* gene alters plasma lipid composition and worsens atherosclerosis in apoE^−/−^ mice. *Sci. Rep.* **7**, 44503; doi: 10.1038/srep44503 (2017).

**Publisher's note:** Springer Nature remains neutral with regard to jurisdictional claims in published maps and institutional affiliations.

## Supplementary Material

Supplementary Information

Supplementary Data S1

Supplementary Data S2

## Figures and Tables

**Table 1:** Plaque size and composition at the aortic sinuses of Ppap2b^f/f^ apoE^−/−^ Alb-Cre^−^ and Ppap2b^f/f^ apoE^−/−^ Alb-Cre^+^ mice.

	Chow diet		Western diet	
	Plpp3^f/f^ apoE^−/−^ Alb-Cre^−^	Plpp3^f/f^ apoE^−/−^ Alb-Cre^+^	Plpp3^f/f^ apoE^−/−^ Alb-Cre^−^	Plpp3^f/f^ apoE^−/−^ Alb-Cre^+^
Lesion size (μm^2^)	494,884 ± 142,501	547,335 ± 150,002	355,308 ± 138,729	479,720 ± 150,857 (**p** = **0.031**)
Necrotic core area (μm^2^)	54,237 ± 23,029	51,201 ± 17,107	40,104 ± 23,571	92,879 ± 35,903 (**p** = **0.0001**)
% total plaque area	11.3 ± 4.3	9.5 ± 2.6	11.0 ± 4.5	19.5 ± 5.1 (**p** = **0.0001**)
Mac-2 positive area (μm^2^)	92,274 ± 53,696	78,784 ± 24,882	147,094 ± 70,170	144,314 ± 49,215
% total plaque area	19.1 ± 8.0	15.8 ± 8.7	41.0 ± 10.0	31.0 ± 8.1 (**p** = **0.0077**)
ORO-positive area (μm^2^)	216,346 ± 73075	226,550 ± 59293	195,505 ± 75,335	232,692 ± 70,157
% total plaque area	44.4 ± 2.8	42.4 ± 9.1	55.9 ± 7.3	49.6 ± 9.8 (**p** = **0.045**)
ECM-positive area (μm^2^)	341,054 ± 96,641	420,028 ± 164,825	180,185 ± 71,971	254,436 ± 104,916
% total plaque area	70.3 ± 14.1	74.5 ± 15.2	50.6 ± 7.4	52.0 ± 10.4
αSMA-positive area (μm^2^)	12,208 ± 4,412	9,569 ± 5,184	12,738 ± 8,922	16,419 ± 6,668
% total plaque area	2.4 ± 0.6	1.9 ± 1.3	3.4 ± 1.2	3.5 ± 1.1
CD3-positive cells (cells/100000 μm^2^)	1.1 ± 0.8	0.6 ± 0.8	3.7 ± 3.4	3.4 ± 3.0

Data are expressed as the mean ± SD; n = 7–8 in Chow diet, n = 16–17 in WD. Mac-2 (macrophages), ORO (Oil Red O, neutral lipids), ECM (extracellular matrix), αSMA (smooth muscle cells), CD3 (T lymphocytes).

**Table 2:** Percentage changes in the overall amounts of lipids in classes caused by hepatic *Plpp3* deletion in mice fed a chow or Western diet.

Total lipids	Chow				Western diet			
	Mean ± SEM (μmol/L)		Mean relative difference (%)	P-value	Mean ± SEM (μmol/L)		Mean relative difference (%)	P-value
	Alb-Cre^+^	Alb-Cre^−^			Alb-Cre^+^	Alb-Cre^−^		
CE	8491 ± 486	8156 ± 509	n.s.	0.863	32812 ± 2757	28131 ± 2238	n.s	0.460
Cer d18:0	0.34 ± 0.02	0.33 ± 0.02	n.s.	0.605	2.2 ± 0.2	1.8 ± 0.1	n.s	0.173
Cer d18:1	9.9 ± 1.1	11.8 ± 1.4	n.s.	0.340	50.1 ± 3.0	50.2 ± 6.9	n.s	0.696
DAG	5.7 ± 0.4	5.9 ± 0.5	n.s.	0.779	22.4 ± 1.2	22.4 ± 2.1	n.s	1.000
Gb3	1.3 ± 0.1	1.1 ± 0.1	n.s.	0.387	4.6 ± 0.2	3.8 ± 0.3	n.s	0.055
Glc/GalCer	28.4 ± 2.3	28.3 ± 2.2	n.s.	1.000	125.3 ± 5.8	113.9 ± 4.8	n.s	0.315
LPA	0.20 ± 0.01	0.19 ± 0.01	n.s.	1.000	0.25 ± 0.02	0.20 ± 0.01	**27%**	**0.034**
LPC	582 ± 30	584 ± 18	n.s.	0.796	1113 ± 29	1059 ± 46	n.s	0.408
LPE	23.0 ± 2.1	21.1 ± 2.0	n.s.	0.436	57.2 ± 4.7	46.9 ± 3.5	n.s	0.146
LPG	9.5 ± 2.8	12.2 ± 4.1	n.s.	0.863	15.8 ± 4.4	12.9 ± 3.6	n.s	0.829
LPI	1.3 ± 0.1	1.2 ± 0.1	n.s.	0.387	3.8 ± 0.2	3.1 ± 0.2	**23%**	**0.016**
LSM	0.006 ± 0.0004	0.006 ± 0.0004	n.s.	0.436	0.014 ± 0.0015	0.012 ± 0.0012	n.s	0.829
LacCer	4.0 ± 0.3	3.0 ± 0.3	**34%**	**0.040**	21.5 ± 1.5	15.9 ± 1.6	**35%**	**0.021**
PC	1328 ± 48	1399 ± 105	n.s.	0.730	3742 ± 160	3573 ± 216	n.s	1.000
PC O	17.2 ± 1.4	14.5 ± 1.3	n.s.	0.161	61.9 ± 2.3	56.9 ± 5.2	n.s	0.360
PE O	2.3 ± 0.2	2.3 ± 0.3	n.s.	0.536	1.4 ± 0.2	1.9 ± 0.3	n.s	0.202
PI	29.0 ± 2.7	29.8 ± 2.3	n.s.	0.796	55.3 ± 2.5	52.2 ± 3.2	n.s	0.829
SM	401 ± 24	378 ± 25	n.s.	0.730	1134 ± 47	981 ± 86	n.s	0.146

The data are expressed as the mean ± SEM; n = 8–10, Wilcoxon rank-sum test. CE = Cholesteryl ester; Cer d18:0 = Ceramide d18:0; Cer d18:1 = Ceramide d18:1; DAG = Diacylglycerol; Gb3 = Globotriaosylceramide; Glc/GalCer = Glucosylceramide/Galactosylceramide; LPA = Lysophosphatidic acid; LPC = Lysophosphatidylcholine; LPE = Lysophosphatidylethanolamine; LPG = Lysophosphatidylglycerol; LPI = Lysophosphatidylinositol; LSM = Lysosphingomyelin; LacCer = Lactosylceramide; PC/PC O = Phosphatidylcholine; PE O = Phosphatidylethanolamine; PI = Phosphatidylinositol; SM = Sphingomyelin.

**Table 3:** Percentage changes in the amounts of lipid species caused by hepatic *Plpp3* deletion in mice fed a chow or Western diet.

		Chow				Western diet			
Lipid		Mean ± SEM (μmol/L)		Mean relative difference (%)	P-value	Mean ± SEM (μmol/L)		Mean relative difference (%)	P-value
class	name	Alb-Cre^+^	Alb-Cre^−^			Alb-Cre^+^	Alb-Cre^−^		
LPA	LPA 0:0/18:1	0.006 ± 0.0005	0.005 ± 0.0007	n.s	0.505	0.012 ± 0.0012	0.007 ± 0.0006	**66%**	**0.009**
LPA	LPA 20:4/0:0	0.019 ± 0.002	0.015 ± 0.002	n.s	0.190	0.035 ± 0.003	0.026 ± 0.002	**31%**	**0.016**
LPC	LPC 0:0/18:2	10.0 ± 0.8	11.0 ± 0.3	**−9%**	**0.040**	5.1 ± 0.3	4.7 ± 0.3	n.s.	0.408
LPE	LPE 18:0	4.3 ± 0.3	3.2 ± 0.3	**36%**	**0.027**	9.5 ± 0.9	8.6 ± 1.0	n.s.	0.633
LPE	LPE 20:3/0:0	0.12 ± 0.02	0.16 ± 0.03	n.s	0.605	0.77 ± 0.14	0.47 ± 0.05	**64%**	**0.043**
LPE	LPE 20:4/0:0	0.59 ± 0.07	0.50 ± 0.09	n.s	0.387	2.19 ± 0.35	1.32 ± 0.14	**66%**	**0.009**
LPE	LPE 22:6/0:0	0.73 ± 0.10	0.79 ± 0.18	n.s	0.863	1.95 ± 0.18	1.43 ± 0.07	**36%**	**0.016**
LPG	LPG 18:1/0:0	0.008 ± 0.001	0.009 ± 0.001	n.s	1.000	0.065 ± 0.004	0.052 ± 0.002	**24%**	**0.012**
LPI	LPI 18:1/0:0	0.025 ± 0.002	0.021 ± 0.002	n.s	0.436	0.400 ± 0.032	0.278 ± 0.012	**44%**	**0.001**
LPI	LPI 18:2/0:0	0.064 ± 0.006	0.049 ± 0.002	**30%**	**0.024**	0.093 ± 0.009	0.072 ± 0.003	n.s.	0.068
LPI	LPI 22:5/0:0	0.007 ± 0.001	0.006 ± 0.001	n.s	0.200	0.033 ± 0.003	0.025 ± 0.002	**32%**	**0.034**
LPI	LPI 22:6/0:0	0.019 ± 0.002	0.017 ± 0.001	n.s	0.666	0.071 ± 0.006	0.054 ± 0.005	**31%**	**0.043**
LacCer	LacCer d18:1/16:0	1.11 ± 0.11	0.80 ± 0.07	**38%**	**0.031**	6.01 ± 0.39	4.6 ± 0.4	**31%**	**0.043**
LacCer	LacCer d18:1/22:0	0.62 ± 0.04	0.52 ± 0.05	n.s	0.113	2.96 ± 0.18	2.28 ± 0.25	**29%**	**0.027**
LacCer	LacCer d18:1/24:0	0.43 ± 0.04	0.35 ± 0.03	n.s	0.222	3.22 ± 0.25	2.31 ± 0.30	**39%**	**0.034**
LacCer	LacCer d18:1/24:1	1.37 ± 0.12	0.97 ± 0.10	**42%**	**0.031**	6.65 ± 0.57	4.64 ± 0.57	**44%**	**0.034**
PC O	PC P-18:0/20:4 (PC O-18:1/20:4)	2.26 ± 0.16	1.57 ± 0.12	**44%**	**0.002**	6.21 ± 0.35	5.38 ± 0.56	n.s.	0.173
SM	SM 42:2	95.4 ± 4.7	91.5 ± 4.3	n.s	0.863	231.9 ± 9	195.4 ± 12.5	**19%**	**0.027**

Data are expressed as mean ± SEM; n = 8–10, Wilcoxon rank-sum test. LPA = Lysophosphatidic acid; LPC = Lysophosphatidylcholine; LPE = Lysophosphatidylethanolamine; LPG = Lysophosphatidylglycerol; LPI = Lysophosphatidylinositol; LacCer = Lactosylceramide; PC O = Phosphatidylcholine; SM = Sphingomyelin.

## References

[b1] Sciorra V. A. & Morris A. J. Roles for lipid phosphate phosphatases in regulation of cellular signaling. Biochim. Biophys. Acta - Mol. Cell. Biol. Lipids 1582, 45–51 (2002). 10.1016/s1388-1981(02)00136-1 12069809

[b2] Sigal Y. J., McDermott M. I. & Morris A. J. Integral membrane lipid phosphatases/phosphotransferases: common structure and diverse functions. Biochem. J. 387, 281–293 (2005). 15801912 10.1042/BJ20041771 PMC1134956

[b3] Kai M., Wada I., Imai S., Sakane F. & Kanoh H. Cloning and characterization of two human isozymes of Mg2+-independent phosphatidic acid phosphatase. J. Biol. Chem. 272, 24572–24578 (1997). 9305923 10.1074/jbc.272.39.24572

[b4] Roberts R., Sciorra V. A. & Morris A. J. Human type 2 phosphatidic acid phosphohydrolases. Substrate specificity of the type 2a, 2b, and 2c enzymes and cell surface activity of the 2a isoform. J. Biol. Chem. 273, 22059–22067 (1998). 9705349 10.1074/jbc.273.34.22059

[b5] Brindley D. N. & Waggoner D. W. Mammalian lipid phosphate phosphohydrolases. J. Biol. Chem. 273, 24281–24284 (1998). 9733709 10.1074/jbc.273.38.24281

[b6] Ren H. . Lipid phosphate phosphatase (LPP3) and vascular development. Biochim. Biophys. Acta - Mol. Cell. Biol. Lipids. 1831, 126–132 (2013). 10.1016/j.bbalip.2012.07.012 PMC3683602 22835522

[b7] Tomsig J. L. . Lipid phosphate phosphohydrolase type 1 (LPP1) degrades extracellular lysophosphatidic acid *in vivo*. Biochem. J. 419, 611–618 (2009). 19215222 10.1042/BJ20081888 PMC2677185

[b8] Zhang N., Sundberg J. P. & Gridley T. Mice mutant for Ppap2c, a homolog of the germ cell migration regulator wunen, are viable and fertile. Genesis. 27, 137–140 (2000). 10992322 10.1002/1526-968x(200008)27:4<137::aid-gene10>3.0.co;2-4

[b9] Escalante-Alcalde D. . The lipid phosphatase LPP3 regulates extra-embryonic vasculogenesis and axis patterning. Development 130, 4623–4637 (2003). 12925589 10.1242/dev.00635

[b10] The Coronary Artery Disease (C4D) Genetics Consortium. A genome-wide association study in Europeans and South Asians identifies five new loci for coronary artery disease. Nat. Genet. 43, 339–344 (2011). 21378988 10.1038/ng.782

[b11] Schunkert H. . Large-scale association analysis identifies 13 new susceptibility loci for coronary artery disease. Nat. Genet. 43, 333–338 (2011). 21378990 10.1038/ng.784 PMC3119261

[b12] Escalante-Alcalde D., Sánchez-Sánchez R. & Stewart C. L. Generation of a conditional Ppap2b/Lpp3 null allele. Genesis. 4, 465–469 (2007). 10.1002/dvg.20314 17610274

[b13] Panchatcharam M. . Lipid phosphate phosphatase 3 negatively regulates smooth muscle cell phenotypic modulation to limit intimal hyperplasia. Arterioscler. Thromb. Vasc. Biol. 33, 52–59 (2013). 23104851 10.1161/ATVBAHA.112.300527 PMC3524385

[b14] Panchatcharam M. . Mice with targeted inactivation of Ppap2b in endothelial and hematopoietic cells display enhanced vascular inflammation and permeability. Arterioscler. Thromb. Vasc. Biol. 34, 837–845 (2014). 24504738 10.1161/ATVBAHA.113.302335 PMC4001868

[b15] Libby P., Ridker P. M. & Hansson G. K. Progress and challenges in translating the biology of atherosclerosis. Nature 473, 317–375 (2011). 21593864 10.1038/nature10146

[b16] Ekroos K., Jänis M., Tarasov K., Hurme R. & Laaksonen R. Lipidomics: a tool for studies of atherosclerosis. Curr. Atheroscler. Rep. 12, 273–281 (2010). 20425241 10.1007/s11883-010-0110-y PMC2878593

[b17] Reis A. . Top-down lipidomics of low density lipoprotein reveal altered lipid profiles in advanced chronic kidney disease. J. Lipid Res. 56, 413–422 (2015). 25424003 10.1194/jlr.M055624 PMC4306694

[b18] Bouwens L., De Bleser P., Vanderkerken K., Geerts B. & Wisse E. Liver cell heterogeneity: functions of non-parenchymal cells. Enzyme 46, 155–168 (1992). 1289080 10.1159/000468782

[b19] Kmieć Z. Cooperation of liver cells in health and disease. Adv. Anat. Embryol. Cell. Biol. 161(**III–XIII**), 1–151 (2001). 10.1007/978-3-642-56553-3 11729749

[b20] Kordes C., Sawitza I., Götze S., Herebian D. & Häussinger D. Hepatic stellate cells contribute to progenitor cells and liver regeneration. J. Clin. Invest. 124, 5503–5515 (2014). 25401473 10.1172/JCI74119 PMC4348953

[b21] Gómez-Muñoz A. Ceramide 1-phosphate/ceramide, a switch between life and death. Biochim. Biophys. Acta. 1758, 2049–2056 (2006). 16808893 10.1016/j.bbamem.2006.05.011

[b22] Titz B. . Effects of cigarette smoke, cessation and switching to two heat-not-burn tobacco products on lung lipid metabolism in C57BL/6 and Apoe−/− mice - an integrative systems toxicology analysis. Toxicol. Sci. 149, 441–457 (2015). 26582801 10.1093/toxsci/kfv244 PMC4725611

[b23] Garner B. . Increased glycosphingolipid levels in serum and aortae of apolipoprotein E gene knockout mice. J. Lipid Res. 43, 205–214 (2002). 11861662

[b24] Bismuth J., Lin P., Yao Q. & Chen C. Ceramide: A common pathway for atherosclerosis? Atherosclerosis 196, 497–504 (2008). 17963772 10.1016/j.atherosclerosis.2007.09.018 PMC2924671

[b25] Bietrix F. . Inhibition of glycosphingolipid synthesis induces a profound reduction of plasma cholesterol and inhibits atherosclerosis development in APOE*3 leiden and low-density lipoprotein receptor−/− Mice. Arterioscler. Thromb. Vasc. Biol. 30, 931–937 (2010). 20167657 10.1161/ATVBAHA.109.201673

[b26] Chatterjee S. . Inhibition of glycosphingolipid synthesis ameliorates atherosclerosis and arterial stiffness in apo E−/− mice and rabbits fed a high fat and cholesterol diet. Circulation 129, 2403–2413 (2014). 24710030 10.1161/CIRCULATIONAHA.113.007559 PMC4053506

[b27] Ichi I. . Association of ceramides in human plasma with risk factors of atherosclerosis. Lipids 41, 859–863 (2006). 17152923 10.1007/s11745-006-5041-6

[b28] Cheng J. M. . Plasma concentrations of molecular lipid species in relation to coronary plaque characteristics and cardiovascular outcome: Results of the ATHEROREMO-IVUS study. Atherosclerosis 243, 560–566 (2015). 26523994 10.1016/j.atherosclerosis.2015.10.022

[b29] Mueller P., Ye S., Morris A. & Smyth S. S. Lysophospholipid mediators in the vasculature. Exp. Cell Res. 333, 190–194 (2015). 25825155 10.1016/j.yexcr.2015.03.016 PMC4408256

[b30] Salous A. K. . Mechanism of rapid elimination of lysophosphatidic acid and related lipids from the circulation of mice. J. Lipid Res. 54, 2775–2784 (2013). 23948545 10.1194/jlr.M039685 PMC3770090

[b31] Knowlden S. & Georas S. N. The autotaxin-LPA axis emerges as a novel regulator of lymphocyte homing and inflammation. J. Immunol. 192, 851–857 (2014). 24443508 10.4049/jimmunol.1302831 PMC3905607

[b32] Dohi T. . Increased circulating plasma lysophosphatidic acid in patients with acute coronary syndrome. Clin. Chim. Acta. 413, 207–212 (2012). 21983165 10.1016/j.cca.2011.09.027

[b33] Kurano M. . Possible involvement of minor lysophospholipids in the increase in plasma lysophosphatidic acid in acute coronary syndrome. Arterioscler. Thromb. Vasc. Biol. 35, 463–470 (2015). 25425621 10.1161/ATVBAHA.114.304748

[b34] Bot M. . Lysophosphatidic acid triggers mast cell-driven atherosclerotic plaque destabilization by increasing vascular inflammation. J. Lipid Res. 54, 1265–1274 (2013). 23396975 10.1194/jlr.M032862 PMC3622323

[b35] Siess W. & Tigyi G. Thrombogenic and atherogenic activities of lysophosphatidic acid. J. Cell. Biochem. 92, 1086–1094 (2004). 15258894 10.1002/jcb.20108

[b36] Navab M. . Source and role of intestinally derived lysophosphatidic acid in dyslipidemia and atherosclerosis. J. Lipid Res. 56, 871–887 (2015). 25646365 10.1194/jlr.M056614 PMC4373744

[b37] Billah M. M. & Lapetina E. G. Formation of lysophosphatidylinositol in platelets stimulated with thrombin or ionophore A23187. J. Biol. Chem. 257, 5196–5200 (1982). 6802848

[b38] Smith D. M. & Waite M. Phosphatidylinositol hydrolysis by phospholipase A2 and C activities in human peripheral blood neutrophils. J. Leukoc. Biol. 52, 670–678 (1992). 1464738 10.1002/jlb.52.6.670

[b39] Bondarenko A. . GPR55-dependent and -independent ion signalling in response to lysophosphatidylinositol in endothelial cells. Br. J. Pharmacol. 161, 308–370 (2010). 20735417 10.1111/j.1476-5381.2010.00744.x PMC2931756

[b40] Piñeiro R. & Falasca M. Lysophosphatidylinositol signalling: new wine from an old bottle. Biochim. Biophys. Acta. 1821, 694–705 (2012). 22285325 10.1016/j.bbalip.2012.01.009

[b41] Oka S., Nakajima K., Yamashita A., Kishimoto S. & Sugiura T. Identification of GPR55 as a lysophosphatidylinositol receptor. Biochem. Biophys. Res. Commun. 362, 928–934 (2007). 17765871 10.1016/j.bbrc.2007.08.078

[b42] Murugesan G. & Fox P. L. Role of lysophosphatidylcholine in the inhibition of endothelial cell motility by oxidized low density lipoprotein. J. Clin. Invest. 97, 2736–2744 (1996). 8675684 10.1172/JCI118728 PMC507366

[b43] Kume N., Cybulsky M. I. & Gimbrone M. A. Lysophosphatidylcholine, a component of atherogenic lipoproteins, induces mononuclear leukocyte adhesion molecules in cultured human and rabbit arterial endothelial cells. J. Clin. Invest. 90, 1138–1144 (1992). 1381720 10.1172/JCI115932 PMC329976

[b44] Kojima Y. Molecular cloning of globotriaosylceramide/CD77 synthase, a glycosyltransferase that initiates the synthesis of globo series glycosphingolipids. J. Biol. Chem. 275, 15152–15156 (2000). 10748143 10.1074/jbc.M909620199

[b45] Skipski V. P. . Lipid composition of human serum lipoproteins. Biochem. J. 104, 340–352 (1967). 6048776 10.1042/bj1040340 PMC1270593

[b46] Bodary P. F., Shayman J. A. & Eitzman D. T. Alpha-galactosidase A in vascular disease. Trends Cardiovasc. Med. 17, 129–133 (2007). 17482095 10.1016/j.tcm.2007.02.006

[b47] Sheppard M. N. The heart in Fabry’s disease. Cardiovasc. Pathol. 20, 8–14 (2011). 19919901 10.1016/j.carpath.2009.10.003

[b48] Shen J. S. . Globotriaosylceramide induces oxidative stress and up-regulates cell adhesion molecule expression in Fabry disease endothelial cells. Mol. Genet. Metab. 95, 163–168 (2008). 18707907 10.1016/j.ymgme.2008.06.016 PMC2593623

[b49] Choi S. . Globotriaosylceramide induces lysosomal degradation of endothelial KCa3.1 in fabry disease. Arterioscler. Thromb. Vasc. Biol. 34, 81–89 (2014). 24158513 10.1161/ATVBAHA.113.302200

[b50] Palinski W. . ApoE-deficient mice are a model of lipoprotein oxidation in atherogenesis. Demonstration of oxidation-specific epitopes in lesions and high titers of autoantibodies to malondialdehyde-lysine in serum. Arterioscler. Thromb. 14, 605–616 (1994). 7511933 10.1161/01.atv.14.4.605

[b51] Parolini C. . A salmon protein hydrolysate exerts lipid-independent anti-atherosclerotic activity in ApoE-deficient mice. PLoS One. 9, 10.1371/journal.pone.0097598 (2014). PMC4026378 24840793

[b52] López-Juárez A. . Expression of LPP3 in Bergmann glia is required for proper cerebellar sphingosine-1-phosphate metabolism/signaling and development. Glia. 59, 577–589 (2011). 21319224 10.1002/glia.21126 PMC3196773

[b53] Gómez-López S. . Neural ablation of the PARK10 candidate Plpp3 leads to dopaminergic transmission deficits without neurodegeneration. Sci. Rep. 6, 24028, 10.1038/srep24028 (2016). 27063549 PMC4827058

[b54] Lee M. R. . The adipokine Retnla modulates cholesterol homeostasis in hyperlipidemic mice. Nat Commun. 5, 4410, 10.1038/ncomms5410 (2014). 25022542

[b55] Marchesi M. . Rosuvastatin does not affect human apolipoprotein A-I expression in genetically modified mice: a clue to the disputed effect of statins on HDL. Br. J. Pharmacol. 164, 1460–1468 (2011). 21486287 10.1111/j.1476-5381.2011.01429.x PMC3221100

[b56] Jung H. R. . High throughput quantitative molecular lipidomics. Biochim. Biophys. Acta. 1811, 925–934 (2011). 21767661 10.1016/j.bbalip.2011.06.025

[b57] Koistinen K. M., Suoniemi M., Simolin H. & Ekroos K. Quantitative lysophospholipidomics in human plasma and skin by LC-MS/MS. Anal. Bioanal. Chem. 407, 5091–5099 (2015). 25618760 10.1007/s00216-014-8453-9

[b58] Ståhlman M. . High-throughput shotgun lipidomics by quadrupole time-of-flight mass spectrometry. J. Chromatogr. B. Analyt. Technol. Biomed. Life. Sci. 877, 2664–2672 (2009). 10.1016/j.jchromb.2009.02.037 19286428

[b59] Ekroos K. . Charting molecular composition of phosphatidylcholines by fatty acid scanning and ion trap MS3 fragmentation. J. Lipid Res. 44, 2181–2192 (2003). 12923235 10.1194/jlr.D300020-JLR200

[b60] Ekroos K., Chernushevich I. V., Simons K. & Shevchenko A. Quantitative profiling of phospholipids by multiple precursor ion scanning on a hybrid quadrupole time-of-flight mass spectrometer. Anal. Chem. 74, 941–949 (2002). 11924996 10.1021/ac015655c

