# Supplementary material for: Liver-specific deletion of the Plpp3 gene alters plasma lipid composition and worsens atherosclerosis in apoE−/− mice
Source: Sci Rep. 2017 Mar 14;7:44503. doi: 10.1038/srep44503 (PMC5349609; doi:10.1038/srep44503)
Supplement: Supplementary Information [file srep44503-s1.pdf]

Liver-specific deletion of the *Plpp3* gene alters plasma lipid composition and worsens atherosclerosis in apoE$^{-/-}$ mice

Marco Busnelli[1^], Stefano Manzini[1^], Mika Hilvo[2], Cinzia Parolini[1], Giulia S. Ganzetti[1], Federica Dellera[1], Kim Ekroos[2], Minna Jänis[3], Diana Escalante-Alcalde[4], Cesare R. Sirtori[1], Reijo Laaksonen[2], Giulia Chiesa[1*]

[1]Department of Pharmacological and Biomolecular Sciences, Università degli Studi di Milano, Milano, Italy; [2]Zora Biosciences Oy, Espoo, Finland; [3]Department of Physiology, Institute of Biomedicine, University of Turku, Turku, Finland; [4]Instituto de Fisiología Celular, División de Neurociencias Universidad Nacional Autónoma de México, Cd. Mx. 04510, México.

^ The Authors contributed equally to this work.

*Corresponding author. Giulia Chiesa, PhD, Department of Pharmacological and Biomolecular Sciences, via Balzaretti, 9, 20133 Milan, Italy; phone: +39-02-50318328, fax: +39-02-50318284; email: giulia.chiesa@unimi.it

Supplementary Figure 1

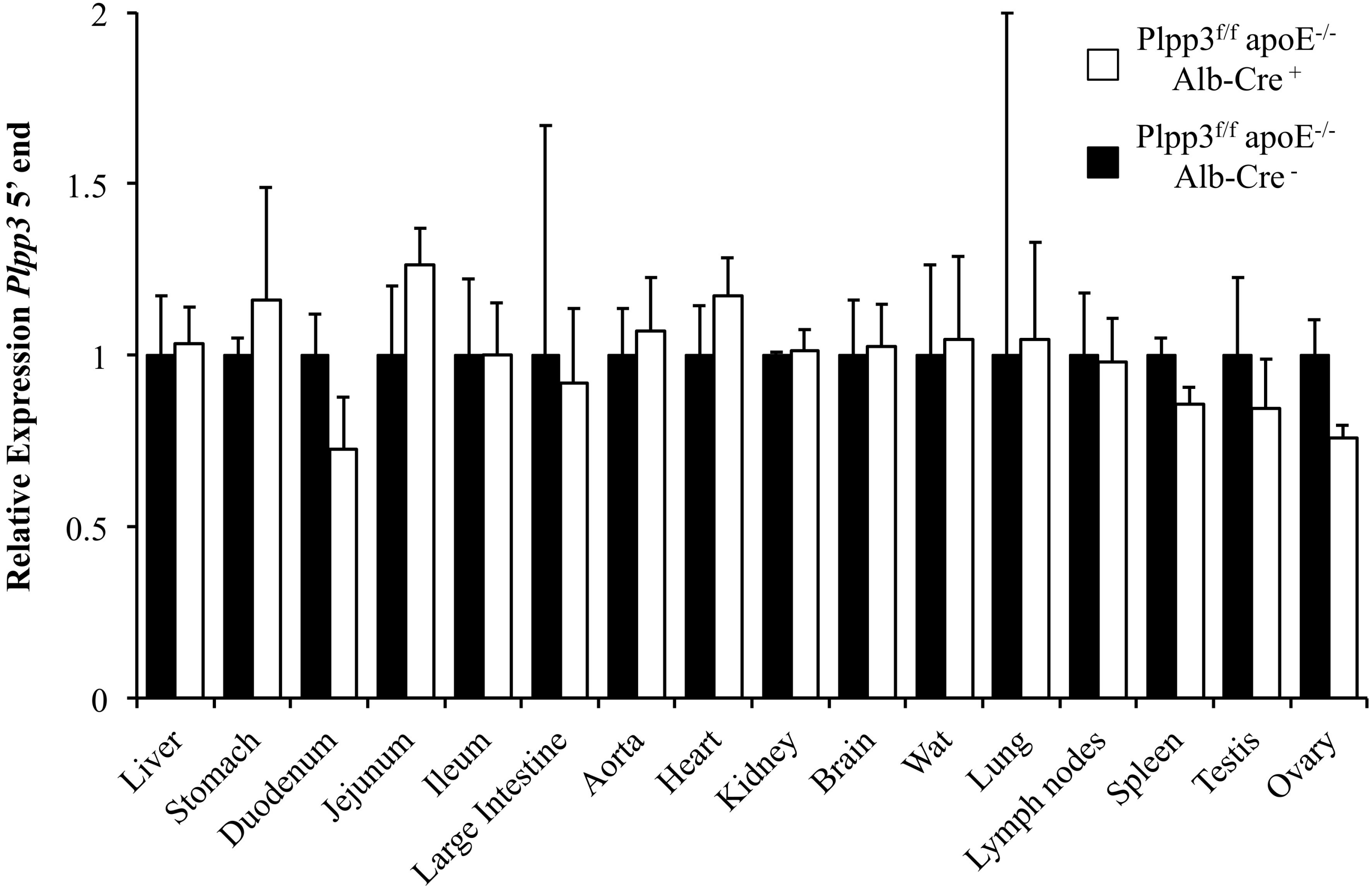

Supplementary Figure 1. Quantitative expression of the *Plpp3* mRNA 5' end. Expression levels of *Plpp3* mRNA are shown, quantified by qPCR targeting the region upstream of the Cre recombinase-mediated floxed exons excision site. Values were normalized to the expression of the transcript in each of the tissues from Plpp3$^{f/f}$ apoE$^{-/-}$ Alb-Cre^{-} mice. (n=6 for liver, n=3 for all the other tissues, $p>0.05$ by Wilcoxon rank-sum test). Wat = white adipose tissue.

Supplementary Figure 2

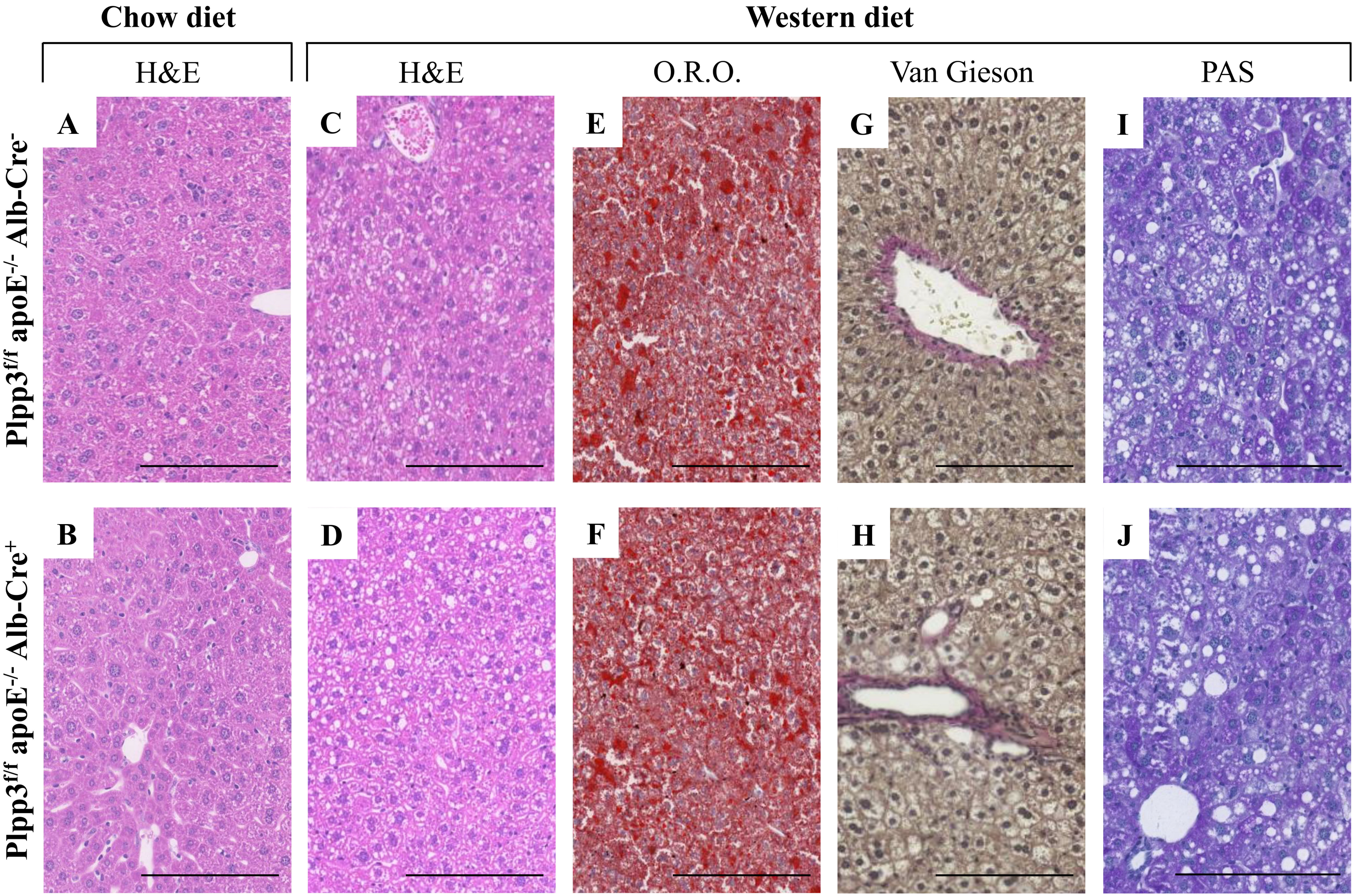

Supplementary Figure 2. Liver histology of chow and Western-diet fed mice. Representative photomicrographs of H&E staining of chow-fed Plpp3$^{f/f}$ apoE$^{-/-}$ Alb-Cre^{-} and Plpp3$^{f/f}$ apoE$^{-/-}$ Alb-Cre^{+} mice (A, B). H&E (C, D), Oil Red O (E, F), Van Gieson, (G, H) and PAS (I, J) staining of liver sections from Western–fed Plpp3$^{f/f}$ apoE$^{-/-}$ Alb-Cre^{-} and Plpp3$^{f/f}$ apoE$^{-/-}$ Alb-Cre^{+} mice. Bar length = 200 µm.

Supplementary Figure 3

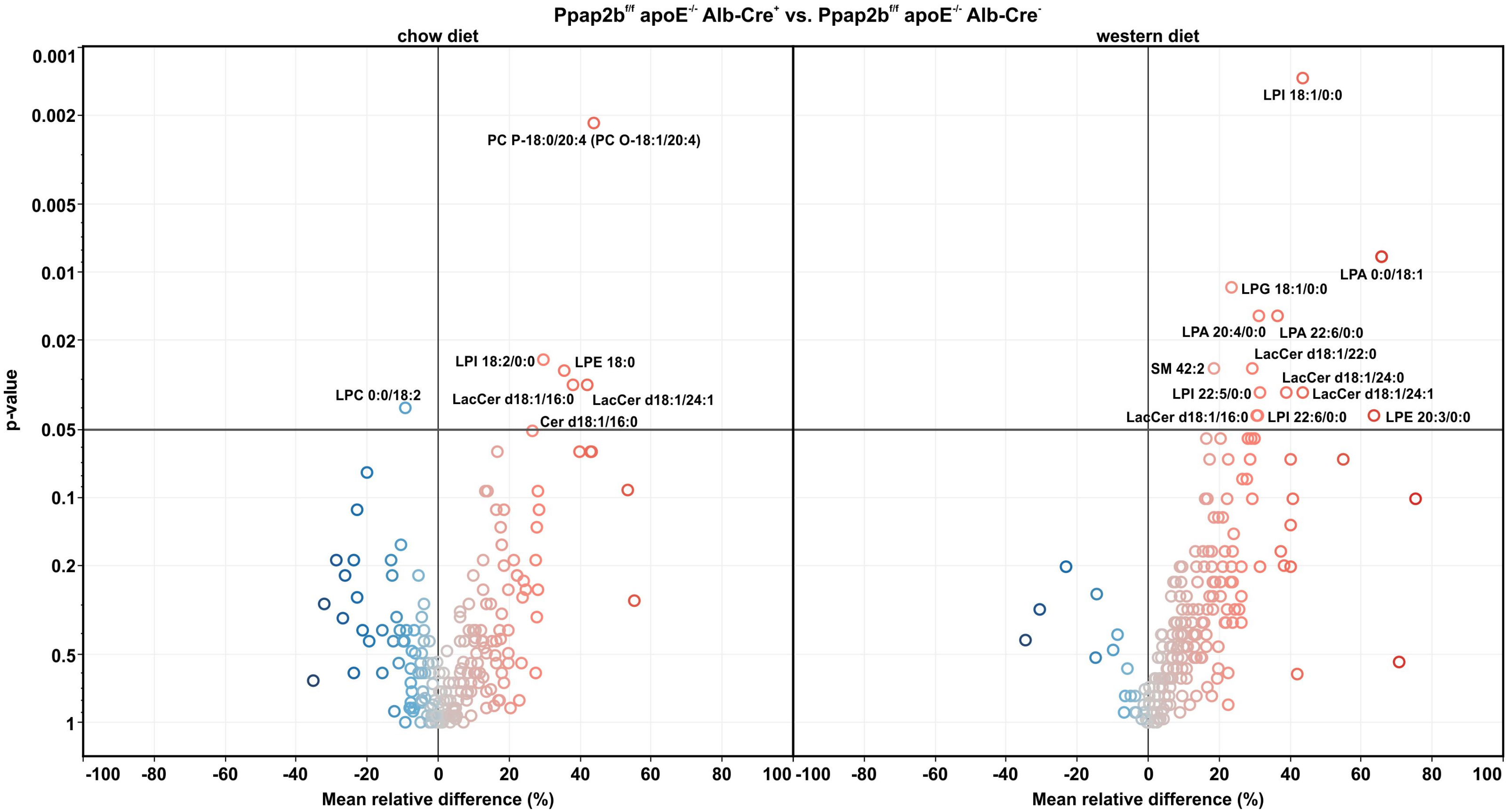

Supplementary Figure 3. Volcano plot highlighting the most significant changes in the levels of plasma lipids caused by hepatic *Plpp3* deletion in mice fed a chow or Western diet. Data are expressed as the percentage of the mean relative difference; n=8-10, Wilcoxon rank-sum test. In the figures, the red colour refers to increased concentrations and the blue colour refers to decreased concentrations in $Plpp3^{f/f}$ $apoE^{-/-}$ $Alb\text{-}Cre^{+}$ vs $Plpp3^{f/f}$ $apoE^{-/-}$ $Alb\text{-}Cre^{-}$ mice. Cer d18:1 = Ceramide d18:1; LacCer = Lactosylceramide; LPA = Lysophosphatidic acid; LPC = Lysophosphatidylcholine; LPE = Lysophosphatidylethanolamine; LPG = Lysophosphatidylglycerol; LPI = Lysophosphatidylinositol; PC O = Phosphatidylcholine; SM = Sphingomyelin.

Supplementary Table 1. Effects of diet on plasma lipid classes in Plpp3$^{f/f}$ apoE$^{-/-}$ Alb-Cre^{-} and Plpp3$^{f/f}$ apoE$^{-/-}$ Alb-Cre^{+} mice.

Total lipids	Plpp3$^{f/f}$ apoE$^{-/-}$ Alb-Cre^{-}				Plpp3$^{f/f}$ apoE$^{-/-}$ Alb-Cre^{+}			
	Mean ± SEM (μmol/L)		Mean relative difference (%)	P-value	Mean ± SEM (μmol/L)		Mean relative difference (%)	P-value
	Western diet	Chow diet			Western diet	Chow diet		
CE	28131 ± 2238	509 ± 8156	**245%**	**8.2E-05**	32812 ± 2757	8491 ± 486	**286%**	**2.2E-05**
Cer d18:0	1.8 ± 0.1	0.02 ± 0.33	**462%**	**8.2E-05**	2.2 ± 0.2	0.34 ± 0.02	**541%**	**2.2E-05**
Cer d18:1	50.2 ± 6.9	1.4 ± 11.8	**327%**	**8.2E-05**	50.1 ± 3	9.9 ± 1.1	**407%**	**2.2E-05**
DAG	22.4 ± 2.1	0.5 ± 5.9	**280%**	**3.1E-04**	22.4 ± 1.2	5.7 ± 0.4	**293%**	**4.6E-05**
Gb3	3.8 ± 0.3	0.1 ± 1.1	**240%**	**8.2E-05**	4.6 ± 0.2	1.3 ± 0.1	**269%**	**2.2E-05**
Glc/GalCer	113.9 ± 4.8	2.2 ± 28.3	**303%**	**8.2E-05**	125.3 ± 5.8	28.4 ± 2.3	**341%**	**2.2E-05**
LPA	0.2 ± 0.01	0.01 ± 0.19	n.s.	**6.7E-01**	0.25 ± 0.02	0.2 ± 0.01	**28%**	**5.7E-03**
LPC	1058.9 ± 46.4	17.9 ± 584.4	**81%**	**8.2E-05**	1113.5 ± 28.9	582 ± 29.5	**91%**	**2.2E-05**
LPE	46.9 ± 3.5	2.0 ± 21.1	**122%**	**5.8E-04**	57.2 ± 4.7	23 ± 2.1	**148%**	**4.3E-05**
LPG	12.9 ± 3.6	4.1 ± 12.2	n.s.	**6.7E-01**	15.8 ± 4.4	9.5 ± 2.8	n.s.	**4.5E-01**
LPI	3.1 ± 0.2	0.1 ± 1.2	**161%**	**8.2E-05**	3.8 ± 0.2	1.3 ± 0.1	**190%**	**2.2E-05**
LSM	0.01 ± 0.001	0.0004 ± 0.0056	**119%**	**5.5E-03**	0.01 ± 0.001	0.0059 ± 0.0004	**130%**	**2.2E-05**
LacCer	15.9 ± 1.6	0.3 ± 3	**438%**	**8.2E-05**	21.5 ± 1.5	4.0 ± 0.3	**441%**	**2.2E-05**
PC	3573 ± 216	105 ± 1399	**155%**	**8.2E-05**	3742 ± 160	1328 ± 48	**182%**	**2.2E-05**
PC O	56.9 ± 5.2	1.3 ± 14.5	**291%**	**8.2E-05**	61.9 ± 2.3	17.2 ± 1.4	**260%**	**2.2E-05**
PE O	1.9 ± 0.3	0.3 ± 2.3	n.s.	**4.4E-01**	1.4 ± 0.2	2.3 ± 0.2	**-37%**	**1.7E-02**
PI	52.2 ± 3.2	2.3 ± 29.8	**75%**	**1.6E-04**	55.3 ± 2.5	29 ± 2.7	**91%**	**4.3E-05**
SM	981 ± 86	25 ± 378	**159%**	**1.6E-04**	1134 ± 47	401 ± 24	**183%**	**2.2E-05**

Supplementary Table 1. Effects of diet on plasma lipid classes in Plpp3$^{f/f}$ apoE$^{-/-}$ Alb-Cre^{-} and Plpp3$^{f/f}$ apoE$^{-/-}$ Alb-Cre^{+} mice. Data are expressed as the mean ± SEM; n=8-10, Wilcoxon rank-sum test. CE = Cholesteryl ester; Cer d18:0 = Ceramide d18:0; Cer d18:1 = Ceramide d18:1; DAG = Diacylglycerol; Gb3 = Globotriaosylceramide; Glc/GalCer = Glucosylceramide/Galactosylceramide; LPA = Lysophosphatidic acid; LPC = Lysophosphatidylcholine; LPE = Lysophosphatidylethanolamine; LPG = Lysophosphatidylglycerol; LPI = Lysophosphatidylinositol; LSM = Lysosphingomyelin; LacCer = Lactosylceramide; PC/PC O = Phosphatidylcholine; PE O = Phosphatidylethanolamine; PI = Phosphatidylinositol; SM = Sphingomyelin.
